# In the Far East

**Published:** 1899-09

**Authors:** 


In the far East it is believed that the child will be exposed to wickedness if its nails are cut during its first year, but after that age regular cutting on Friday preserves it from toothache.

